# Cellular microenvironment modulates the galvanotaxis of brain tumor initiating cells

**DOI:** 10.1038/srep21583

**Published:** 2016-02-22

**Authors:** Yu-Ja Huang, Gwendolyn Hoffmann, Benjamin Wheeler, Paula Schiapparelli, Alfredo Quinones-Hinojosa, Peter Searson

**Affiliations:** 1 Institute for Nanobiotechnology, Johns Hopkins University, Baltimore, Maryland, United States of America; 2 Department of Materials Science and Engineering, Johns Hopkins University, Baltimore, Maryland, United States of America; 3 Department of Neurosurgery and Oncology, Sidney Kimmel Comprehensive Cancer Center, Johns Hopkins University, Baltimore, Maryland, United States of America

## Abstract

Galvanotaxis is a complex process that represents the collective outcome of various contributing mechanisms, including asymmetric ion influxes, preferential activation of voltage-gated channels, and electrophoretic redistribution of membrane components. While a large number of studies have focused on various up- and downstream signaling pathways, little is known about how the surrounding microenvironment may interact and contribute to the directional response. Using a customized galvanotaxis chip capable of carrying out experiments in both two- and three-dimensional microenvironments, we show that cell-extracellular matrix (ECM) interactions modulate the galvanotaxis of brain tumor initiating cells (BTICs). Five different BTICs across three different glioblastoma subtypes were examined and shown to all migrate toward the anode in the presence of a direct-current electric field (dcEF) when cultured on a poly-L-ornithine/laminin coated surface, while the fetal-derived neural progenitor cells (fNPCs) migrated toward the cathode. Interestingly, when embedded in a 3D ECM composed of hyaluronic acid and collagen, BTICs exhibited opposite directional response and migrated toward the cathode. Pharmacological inhibition against a panel of key molecules involved in galvanotaxis further revealed the mechanistic differences between 2- and 3D galvanotaxis in BTICs. Both myosin II and phosphoinositide 3-kinase (PI3K) were found to hold strikingly different roles in different microenvironments.

Glioblastoma (GBM) is among the most aggressive types of cancer with a median survival time only slightly more than a year following diagnosis^1^. Malignant glioma cells tend to migrate along blood vessels in the perivascular space or the white matter tracks within the brain parenchyma^2^. The diffusive nature of invasion imposes a major challenge in the treatment of glioblastoma. An emerging strategy for treatment focuses on the subpopulation of brain tumor initiating cells (BTICs) residing in the perivascular niche that are capable of self-renewal and differentiation^3^. Understanding how various chemical and physical signaling pathways regulate the functionality and invasion of BTICs can lead to better treatment strategies against glioblastoma.

Glioblastoma cells are known to respond to various migration cues. Chemokines such as bradykinin, EGF and PDGF induce directional migration via chemotaxis, whereas physical parameters such as interstitial flow and contact guidance can also mediate invasion of human BTICs^4^. More recently, a direct current electric field (dcEFs) of 0.03 V cm^−1^ was measured between the subventricular zone and olfactory bulb in the mouse brain and was suggested as a driving force to direct the migration of neuroblasts along the rostral migration stream (RMS)^5^. The existence of an RMS-like pathway both in fetal and adult human brains has been recently reported^6^ although the existence and magnitude of a local EF remains to be established. BTICs may be derived from adult neural stem cells, multipotent neural progenitor cells (NPCs), or astrocytes^7^. Evidence suggests that both GBM cells, such as BTICs, and NPCs migrate along microvessels and nerve bundles in the extracellular space^2^. Taken together these results suggest that endogenous EFs may influence the migration of BTICs and NPCs in the brain. Understanding and controling the directional migration of BTICs may ultimately lead to new therapies.

Numerous cell types of different origins were previously shown to migrate either toward the cathode or anode in the presence of a dcEF, a process known as galvanotaxis^8^. The precise mechanisms for galvanotaxis are still largely unknown but are thought to involve asymmetric ionic flow through various voltage-gated channels^8^ and electrophoretic redistribution of charged membrane components^9^. To understand whether a dcEF is a potent migration cue for the invasion of glioblastoma and whether the driving mechanism is different from other cell types, a chip-based galvanotaxis device capable of long-term observation was constructed using microfabrication (Fig. 1). GBM can be classified into four different subtypes based on gene expression-based molecular classifications^10^. Here we examined the galvanotaxis of five different patient-derived GBM cell lines across three GBM subtypes and compared them with the responses seen in immortalized GBM cells (U87) and fetal-derived neural progenitor cells (fNPCs). We show that while U87 cells did not possess any directional bias in the presence of a 1V cm^−1^ EF, all primary GBM cell lines exhibited strong anodic responses on a 2D surface coated with ornithine and laminin, in contrast to the cathodic response seen in fNPCs. The device was further optimized to study galvanotaxis in a 3D ECM as it provides a more physiological relevant environment. By directly comparing 2- and 3D galvanotaxis, we show significant phenotypic and mechanistic differences between two different microenvironments. In addition to the opposite directional responses, the roles of myosin II and phosphoinositide 3-kinase (PI3K) were also drastically different in 2D and 3D. We highlight here the complexity of galvanotaxis and show that galvanotaxis is not only cell-type specific but is also greatly influenced by cell-ECM interactions.

## Materials and Methods

### Equipment and reagents

An inverted microscope (Nikon, Eclipse TiE) equipped with a confocal laser module and a live-cell chamber was used for imaging. Electric fields were applied via a potentiostat (Princeton Applied Research, VersaStat 3, Oak Ridge, TN) operating in a constant voltage mode while monitoring the current supplied. BTIC medium was prepared from DMEM/F12 (Invitrogen, US) with B-27 supplements (Invitrogen, US), hEGF (20 ng mL^−1^, Peprotech, Rocky Hill, NJ) and hFGF (20 ng mL^−1^, Peprotech, Rocky Hill, NJ). Poly-L-ornithine and laminin were purchased from Sigma Aldrich (St. Louis, MO). Type I rat tail collagen was purchased from Corning (Tewksbury, MA) and hyaluronic acid (Hystem) was from Sigma Aldrich. Latrunculin A, nocodazole, and LY294002 were obtained from Sigma; all other chemical inhibitors were purchased from Tocris Bioscience. Primary antibodies for α-tubulin (Abcam), phalloidin, and DAPI (Life Technology) were used at the concentration recommended by the manufacturer for immunofluorescence.

### Cell lines

Early passages of human brain tumor initiating cells (BTICs), were used and previously validated by Johns Hopkins Genetic Resources Core Facility^11^. GBM612 cells isolated from intraoperative tissue are multipotent and are able to form diffuse tumors when implanted into animal models^11^,^12^,^13^. BTICs were grown in culture flasks coated with poly-L-ornithine and laminin and cultured with stem cell media composed of DMEM/F12, B27 supplements, EGF, and FGF^14^. The molecular subtypes of the GBM cell lines were characterized previously using a metagene score based approach^15^. Three different GBM subtypes including proneural (GBM 612 and 276), mesenchymal (GBM 626 and 253), and classical (GBM 965) were selected for this study. U87 cells were acquired from ATCC and cultured as recommended in EMEM supplemented with 10% fetal bovine serum. Primary fetal neural progenitor cells F54 were obtained as described previously^16^ and were maintained in 2:1 high-glucose DMEM (Invitrogen)/Ham’s F-12 (Cellgro), 1X B-27, 1% anti–anti, 20 ng/mL bFGF, 20 ng/mL EGF, 20 ng/mL leukemia inhibitory factor (LIF, Millipore, Billerica, MA), and 5 μg/mL heparin (Sigma).

### Two-dimensional galvanotaxis and cell tracking

**Figure 1:**
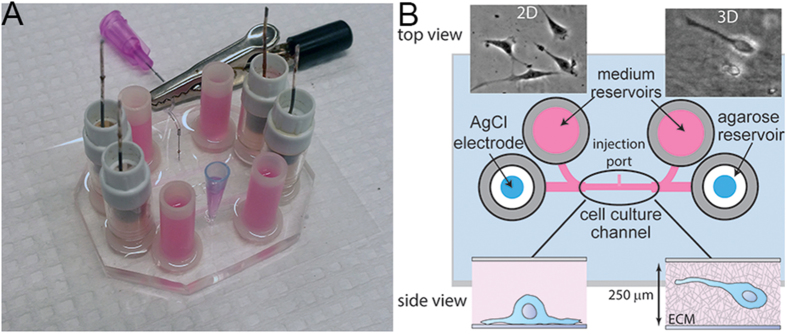
A chip-based device for studying galvanotaxis in 2D and 3D. (**A**) The galvanotaxis chamber is attached to a 35 mm × 50 mm glass coverslip after treated with oxygen plasma. Each chip contains two measurement channels. (**B**) Schematic illustration of the galvanotaxis device. Each device contains two coiled Ag/AgCl electrodes embedded in agarose reservoirs and two media reservoirs separated by a cell culture channel in the middle. For 2D studies, cells are seeded on a PLO/LN coated surface via the cell injection port using a syringe. For 3D studies, cells embedded in an ECM gel were introduced into the channel via a pipette tip to minimize the formation of bubbles at the injection port. The dimensions of the cell culture channel are 10 mm × 5 mm × 250 μm (L×W×H).

Galvanotaxis experiments were carried out using a customized galvanotaxis device reported previously, utilizing standard microfabrication techniques (Fig. 1A)^17^. Briefly, BTICs were seeded in a central channel coated with PLO/LN for 24 hours before mounting onto a microscope equipped with a live-cell chamber for time-lapse experiments. In each experiment, an electric field was applied through a pair of Ag/AgCl electrodes embedded in agarose via a potentiostat operated in a constant-voltage mode. Cells were stimulated in a dcEF for 3–9 hours before being fixed for immunofluorescence studies.

The trajectories of cells from time-lapse images were automatically tracked using Metamorph (Molecular Devices, US) to minimize any tracking biases. Only isolated cells that remained in the field of view and did not undergo mitosis were selected for analysis. Cell trajectories were further analyzed using a customized Matlab (MathWorks, US) script to characterize physical parameters including cell motility and directedness. Here we define cell motility as the total path length traveled by a cell divided by the elapsed time. The directedness is defined as Σcosθ_i_/n, where n is the total number of cells and θ_i_ is the angle between the vector of cell displacement and electric field vector^17^.

### Drug inhibition assay

For each drug inhibition study, cells were treated with the compound at the indicated concentration for at least three hours before experiments. The molecules studied were: Latrunculin A (250 ng mL^−1^), nocodazole (200ng mL^−1^), blebbistatin (10 μM), ZCL278 (50 μM), NSC23766 (50 μM), Y27632 (50 μM), LY294002 (50 μM), U0126 (10 μM), SB202190 (10 μM), PD158780 (10 μM), SU5402 (25 μM), Imanitib (1 μM), AMD3100 (50 μM), and SB225002 (1 μM).

### Immunofluorescence imaging

BTICs were fixed with 3.7% formaldehyde, permeabilized, and stained following standard procedures. Primary antibodies were used at a 1:100 dilution and incubated overnight at 4 °C. Secondary antibodies conjugated to fluorophores were used at a 1:200 dilution and incubated for an hour before washing and imaging. Immunostaining of neurospheres for stem cell markers were carried out following procedures reported previously^18^.

### Galvanotaxis of BTICs in a three-dimensional matrix

BTICs were embedded in a matrix composed of a mixture of type I collagen and hyaluronic acid and stimulated with a dcEF to study migration in 3D. Briefly, approximately 10^5^ BTICs were mixed with 100  μL of a mixture of 1 mg mL^−1^ of type I collagen and 1 mg mL^−1^ of hyaluronic acid and introduced into the cell culture channel at the cell injection port via a pipette tip (Fig. 1A). The gel composition was previously optimized to recapitulate glioma invasion *in vitro* and has a comparable stiffness to the brain ECM^4^. The cell/ECM mixture was allowed to polymerize for 30 minutes at 37 °C before 100  μL of medium was added into both media reservoirs to keep the gel hydrated. The final dimensions of the gel were defined by the channel size and measured to be 10 mm × 5 mm × 250 μm (L×W×H). Cells were allowed to spread for at least 36 hours before mounting onto the microscope for time-lapse experiments. Only cells that were embedded in the center of the gel, at least about 100 μm from the side wall, and remained within the same focal plane throughout the designated time were tracked and analyzed.

## Results and Discussion

### BTICs cultured on a poly-L-ornithine/laminin surface migrate toward the anode in a voltage dependent manner

**Figure 2:**
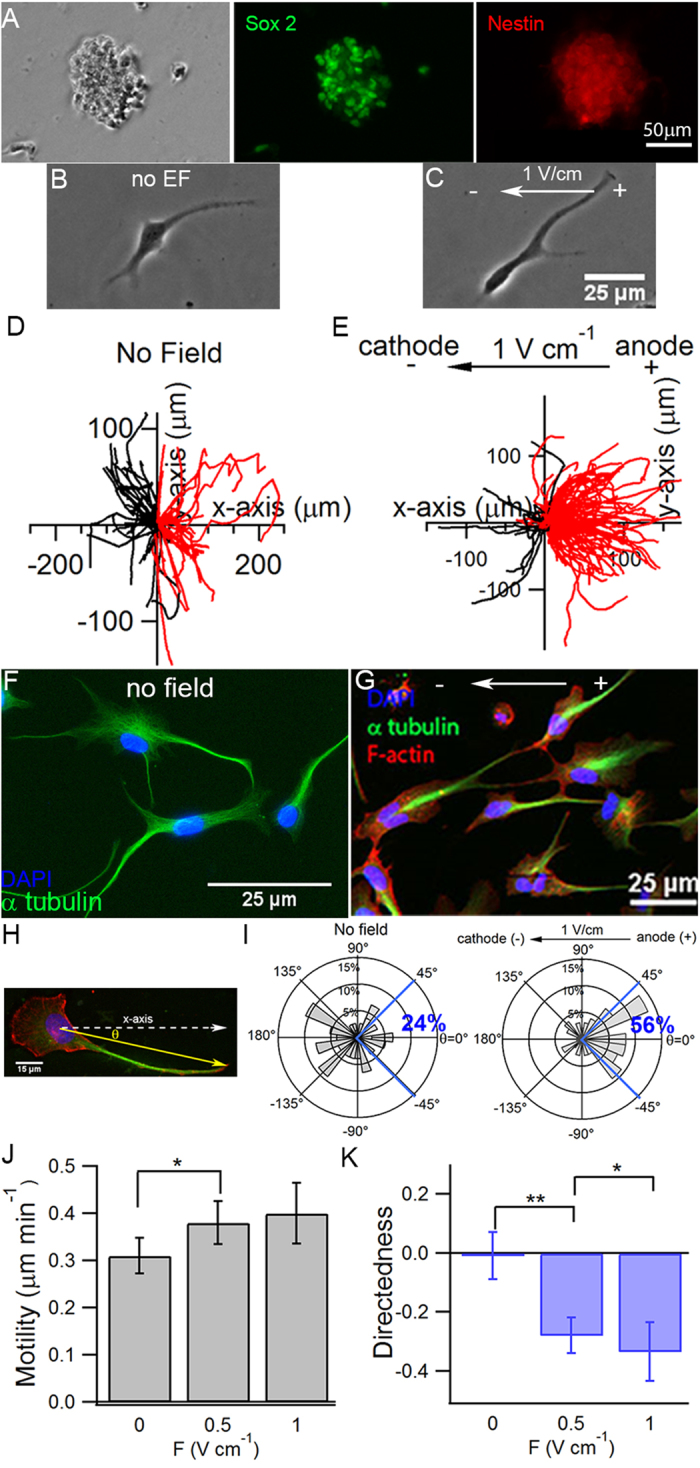
Characterization of the galvanotaxis of BTICs in 2D. (**A**) GBM612 cells clustered into neurospheres when cultured in suspension and stained positive for stem cell markers Sox 2 and nestin. (**B**) Prior to each experiment, BTICs were seeded as single cells to characterize their migration. (**C**) Representative image of a BTIC in the presence of an EF after one hour; the cell extended a characteristic protrusion toward the anode. Overlay of the trajectories in the absence (**D**) and presence (**E**) of a dcEF show the anodic response of BTICs in the presence of an EF. Each trajectory represents the actual path traveled by a cell in 3 hours either to the cathode (left, black) or anode (right, red). Cells stained for microtubules in the absence of an EF (**F**) showed random orientation of protrusions whereas cells in the presence of an EF (**G**) extended its protrusions toward the anode; overlaying microtubules with actins shows that microtubules were found in the center whereas actin filaments were largely at the periphery of a protrusion. (**H**) The protrusion angle (θ) is defined as the angle between the protrusion vector and the positive x-axis, where −180° ≤ θ ≤ 180°.The protrusion vector is defined as the vector originating from the center of the nucleus to the end of the longest cell protrusion. (**I**) Distributions of θ in the absence (left) and presence (right) of an EF show that an applied electric field induced formations of protrusions toward the anode. (**J**) Cell motility increased in the presence of a dcEF. Further increasing the EF from 0.5 V cm^−1^ to 1V cm^−1^ did not significantly increase cell motility. (**K**) An electric field as small as 0.5 V cm^−1^ was capable of biasing cell trajectories toward the anode. Cell directedness further increased with increasing electric field. *P < 0.05; **P < 0.01; Student’s t-test. Statistics were obtained from at least three independent experiments with at least 60 cells in each experiment. Error bars represent standard deviation.

To characterize the galvanotaxis of BTICs, GBM612 cells (Fig. 2A) were cultured and stimulated with a direct-current electric field (dcEF) using our chip-based platform (Fig. 1A,B). In the absence of a dcEF, GBM612 cells migrated in a non-directional manner (Fig. 2D) with a motility of 0.31 ±  0.04 μm min^−1^ and a mean directedness of −0.01 ± 0.08. However, in the presence of an EF, the cell trajectories were significantly biased toward the anode (Fig. 2E, Supplementary Video S1). In response to the dcEF, BTICs also oriented with their long axis in the direction of electric field and extended protrusions toward the anode (Fig. 2C). Immunostaining in cells under no EF (Fig. 2F) or a 1V cm^−1^ dcEF (Fig. 2G) showed that cell protrusions were abundant with microtubules and highly oriented toward the anode in the presence of an EF. By analyzing the angle (θ) between a cell’s protrusion vector and the positive x-axis (Fig. 2H), we showed that in the presence of an EF, 56% of cells had average protrusion angles between ±45°, indicating preferential alignment whereas, in the absence of an EF, only 24% of cells had average protrusion angles within this range (Fig. 2I). A dcEF as small as 0.5 Vcm^−1^ was capable of significantly increasing cell motility and biasing cell trajectories toward the anode (Fig. 2J and K). Further increasing the electric field to 1V cm^−1^ did not further enhance cell motility (0.41 ± 0.07 μm min^−1^), but further biased cell trajectories toward the anode with a mean directedness of−0.47 ± 0.12 (p < 0.01) (Fig. 2K).

### GBM cells of different subtypes migrate toward the anode, while neural progenitor cells (NPCs) migrate toward the cathode

**Figure 3:**
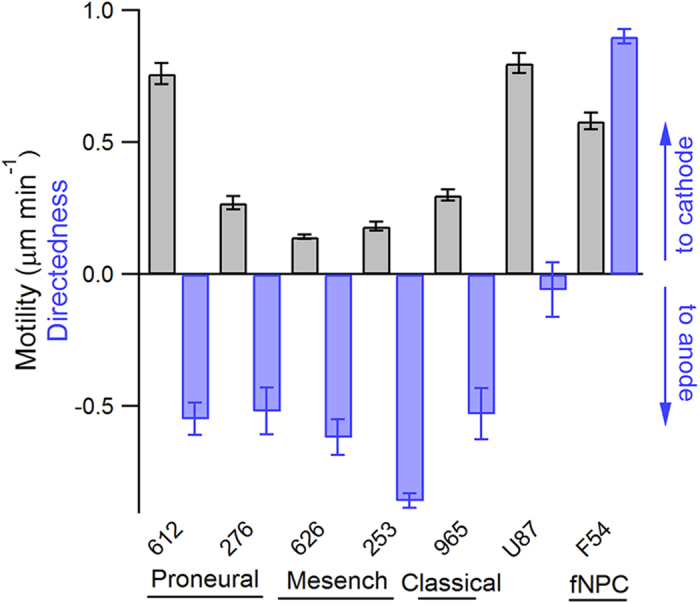
Summary of the galvanotaxis of GBM cells and fNPCs. Three out of four GBM subtypes were tested for galvanotaxis, where cells belong to the proneural (GBM 612 and 276), mesenchymal (GBM 626 and 253), and classical subtypes (GBM 965) all migrated toward the anode in the presence of a 1V cm ^−1^ EF. The immortalized U87 GBM cell line exhibited no directional bias. Fetal-derived NPCs, however, migrated toward the cathode with high directedness in an EF, opposite to primary GBM cells. At least 50 cells per experiment were analyzed and reported. Data are shown as mean ± SEM.

To assess whether the response of the GBM612 cells is the same in other cell types, we studied five different patient-derived GBM cell lines across three different subtypes, as well as an immortalized GBM cell line (U87), and fetal-derived neural progenitor cells (fNPCs). Interestingly, in the presence of an EF, all primary proneural, mesenchymal, and classical GBM subtypes migrated toward the anode on a PLO/LN coated surface (Fig. 3 and Supplementary Fig. S1). U87, a commonly used immortalized cell line to study GBM, however, exhibited no directional bias in the presence of a 1V cm^−1^ EF (d = −0.06) (Fig. 3 and Supplementary Fig. S1). Although superoxide has been shown to mediate the galvanotaxis of U87 cells toward the cathode at 2 V cm^−1^^19^, we suspected the lack of response we observed here is due to a weaker applied EF. Increase the EF to 5 V cm^−1^ did induce a cathodic response in U87 cells (d = 0.5, at 5 V cm^−1^). Lastly, since neural progenitor cells are considered a target for malignant transformation into brain tumor stem cells^20^, fNPCs were also tested for galvanotaxis. Strikingly, fNPCs exhibited a strong cathodic response even under a weak EF (d = 0.91, Fig. 3 and Supplementary Fig. S1) similar to a previous study^21^. fNPCs also displayed a drastically different phenotype compared to BTICs. In the presence of a 1V cm^−1^ EF, fNPCs oriented perpendicular to the EF and extended broad fan-like lamellipodia while migrating toward the cathode with high persistence (Supplementary Video S2 and Supplementary Fig. S2A). Given the large differences in sensitivity and directional response among primary tumorigenic GBM cells, immortalized GBM cells, and fetal-derived NPCs, we suspected the anodic response acquired by primary GBM cells is a consequence of pathogenesis. GBM 612 cells were further selected to investigate the mechanistic details of galvanotaxis due to their high motility and directional response.

### Galvanotaxis of BTICs is not solely dependent on small-GTPases

**Figure 4:**
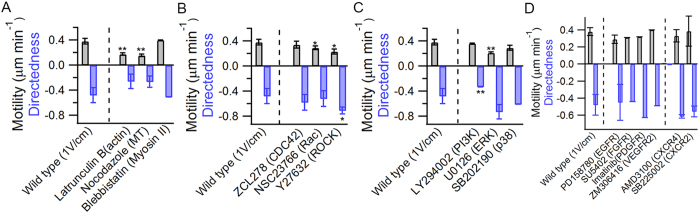
Drug screening assay reveals galvanotaxis of BTICs in 2D is negatively regulated by ROCK and MAPK but dependent on the activation of PI3K. (**A**) Effective galvanotaxis involves both actin and microtubules. Inhibition of actin filaments or microtubules significantly down-regulated cell motility without abolishing the directional response, whereas myosin II had no effect on either motility or directedness. (**B**) Blocking either Rac or ROCK down-regulated cell motility (p = 0.016 and 0.03 respectively), whereas inhibiting Cdc42 did not have a significant effect on galvanotaxis. Inhibition of ROCK also significantly increased directedness of cells (p = 0.017). (**C**) The anodic response of BTICs is regulated by PI3K but negatively regulated by Erk. Disruption of PI3K significantly down-regulated directedness (p = 0.009) but had no effect on cell motility, whereas inhibiting Erk down-regulated motility but increased directedness (p = 0.11). (**D**) Pharmacological perturbations of known receptors involved in the chemotaxis of glioblastoma including EGFR, FGFR, PDGFR, VEGFR2, CXCR4 and CXCR2 had no effect on either cell motility or directedness. Note: all experiments were conducted at 1V cm^−1^. *P < 0.05; **P < 0.01; Student’s t-test. Statistics were obtained by comparing the drug treatment group to the control (no drug). Each condition reported consists of at least two independent experiments with at least 50 cells in each experiment except for the inhibition of FGFR, PDGFR, and VEGFR2. Error bars indicate standard deviation.

The Rho family of GTPases, such as Cdc42, Rac, and Rho, are known to relay external signals and regulate actin cytoskeleton and microtubule dynamics that are important for effective long range cell migration^22^. Inhibiting actin polymerization with 250 ng mL^−1^ of latrunculin B or microbubules with 200 ng mL^−1^ of nocodazole significantly attenuated cell motility (Fig. 4A). However, despite inhibition of actin polymerization and microtubule disruption, cell migration still remained biased toward the anode, indicating that directional field sensing can exist without feedback from dynamic cell migration, similar to chemotaxis^23^. On the other hand, inhibiting the activity of myosin II with 10 μM blebbistatin had no effect on either cell motility or directedness, which suggests that cells were able to adapt to the traction loss due to the inhibition of myosin II, similar to a previous study^24^.

Next, BTICs were treated with Rho GTPase inhibitors to investigate their involvement in galvanotaxis. Selective inhibition of Cdc42 with 50 μM of ZCL278 had no effect on either cell motility or directedness (Fig. 4B). In contrast, 50 μM of ZCL278 has been shown to suppress the formation of filopodia and significantly suppress cell migration into the wound area in a human prostate cancer cell line PC-3^25^. One possible explanation for this discrepancy is that directional sensing in galvanotaxis is a physical process, where electrophoretic or electroosmotic forces provide physical guidance cues, similar to the response of cells to shear stress^26^. For example, shear stress-induced polarization of endothelial cell was shown to be mediated by Rac and Rho, but not Cdc42 or PI3-kinases^26^. Disruption of Rac1 with 50 μM of NSC23766 or Rho-associated protein kinase (ROCK) with 50 μM of Y27632 significantly downregulated cell motility to 0.31 (p = 0.046) and 0.26 μM min^−1^ (p = 0.03), respectively (Fig. 4B). Although perturbations of Rac1 had no effect on directedness, cells treated with ROCK inhibitor showed an unexpected increase in directedness (d = −0.71, p = 0.02) (Supplementary Video S3). These results are in contrast to studies of human keratinocytes, where inhibiting Rac1 completely abolished cathodic galvanotaxis^27^, and in human induced pluripotent stem cells where inhibiting ROCK significantly increased cell motility but reduced the anodic directional response by 70–80%^28^. However, ROCK has been found to spatially regulate the activation of myosin light chains in fibroblasts and inhibiting ROCK increased cell persistence^29^, consistent with the increase in directedness shown here (Fig. 4B).

### Inhibition of PI3-K attenuated directional response without compromising cell motility

Phosphatidylinositol-3-kinase (PI3K) has been implicated in several previous studies of galvanotaxis^30^,^31^,^32^,^33^. Electrical migratory cues are thought to be relayed through the Src/PI3K/Akt and MAPK signaling pathways by cytoskeleton rearrangement that ultimately results in directional migration^30^. However, the role of PI3K is dependent on cell type: inhibiting PI3K significantly impaired the speed and directedness of neural progenitor cells and keratocytes^9^,^31^ and abolished the pre-angiogenic response in endothelial cells^34^, but only had a slight effect on breast cancer cells^32^.

BTICs were incubated with 50 μM of LY294002 before applying a dcEF. In the presence of a 1V cm^−1^ dcEF, cells treated with PI3K inhibitor showed similar motility (0.36 μm min^−1^) to the control group with a moderate but statistically significant decrease in directional response (directedness, d = −0.32 compared to d = −0.48 for control, p = 0.009) (Fig. 4C, Supplementary Video S4). However, inhibited cells remained capable of sensing and migrating toward the anode, indicating that there are parallel mechanisms contributing to the galvanotaxis of BTICs. Similar results were obtained at a concentration of 200 μM of LY294002. Crosstalk between PI3K and MAPK pathways has been implicated in the maintenance of self-renewal and tumorigenicity of glioblastoma stem-like cells^35^. Since both pathways have been implicated in galvanotaxis^8^,^36^, we suspected the involvement of MAPK signaling in galvanotaxis.

### Inhibition of Erk increased cell directedness

Mitogen activated protein kinases (MAPKs), including Erk, p38, and JNK, are involved in migration, apoptosis, and inflammation^37^. Erk1/2 has been implicated in the galvanotaxis of bovine corneal epithelial cells and breast cancer cells via the asymmetric upstream activation of EGFR^32^,^36^. Inhibiting Erk1/2 significantly down regulated both the motility and directedness in corneal epithelial cells and breast cancer cells even though the former migrated toward the cathode and the latter migrated toward the anode^32^,^36^. The roles of p38 and JNK in galvanotaxis have not been explored. Inhibiting Erk1/2 using 10 μM of U0126 significantly down regulated the motility of BTICs but increased their directedness in the presence of an electric field (Fig. 4C), similar to the effect of blocking ROCK, where cells have significantly lower motility (0.21 μm min^−1^, p < 0.01) but increased directedness (d = −0.73, p = 0.11, Supplementary Video S5). On the other hand, inhibiting p38 MAPK with 10 μM SB202190 had no effect on either cell motility or directedness (Fig. 4C). Since ROCK was previously shown to contribute to the phosphorylation of Erk1/2 in glioblastoma cells^38^, one possibility to explain the observed results could be that cross-talk between ROCK and Erk in the presence of a dcEF promoted cell motility via its downstream myosin light chain kinase^39^. However, it may be equally likely that ROCK and Erk acted on galvanotaxis of BTICs via independent pathways. Since Erk1/2 are known to be activated by various growth factors, such as EGF, FGF, PDGF and VEGF, we then targeted receptor tyrosine kinases (RTKs) located on the cell membrane to further stratify the mechanisms of galvanotaxis.

### EGFR, FGFR, PDGFR, and VEGFR2 are not involved in the galvanotaxis of BTICs

Phosphorylation of receptor tyrosine kinases (RTKs) is known to activate the downstream signaling cascade of PI3K/Akt and MAPK pathways^30^,^31^,^32^,^33^. Possible RTKs that may be involved in the galvanotaxis of BTICs include epidermal growth factor receptor (EGFR), fibroblast growth factor receptor (FGFR), platelet derived growth factor receptor (PDGFR), and vascular endothelial growth factor receptor (VEGFR), as each RTK is known to regulate cancer invasion via chemotaxis^40^. Epidermal growth factor receptors (EGFRs) have been shown to be polarized at the leading edge of migrating epithelial cells in the presence of an electric field and asymmetrically activated downstream MAP kinase ERK1/2^41^. Inhibition of EGFR significantly diminished the directedness and motility of galvanotaxis in neural progenitor cells^21^. An applied dcEF also induced pre-angiogenic response in vascular endothelial cells through PI3K-Akt and Rho-ROCK signaling pathways^34^. Yet, the involvement of FGFR and PDGFR in galvanotaxis have not been reported. We found that pharmacological inhibition of EGFR (10 μM PD158780), FGFR (25 μM SU5402), PDGFR (1 μM imanitib), or VEGFR2 (10 μM ZM306416) in the presence of a 1V cm^−1^ dcEF had no effect on either cell motility or directedness (Fig. 4D). These results suggest that the galvanotaxis of BTICs is unlikely triggered by the activation of MAPKs via asymmetric signaling of RTKs.

### CXCR4 and CXCR2 are not involved in galvanotaxis

CXC chemokine receptors (CXCRs) belong to a subfamily of G-protein coupled receptors that are highly involved in cell motility and survival^42^. Among the CXCR family, CXCR4 and CXCR2 were previously reported to modulate the invasion of glioma cells^43^,^44^. In addition, CXCR4 is also a key receptor in chemotaxis of many types of stem cells^45^,^46^. Since inhibiting PI3K attenuated the directedness of BTICs, we wondered whether galvanotaxis was first initiated by either CXCR4 or CXCR2 and which then triggered the activation of PI3K. However, treating cells with either 50 μM of AMD3100, an antagonist for CXCR4, or 1 μM of SB225002, an antagonist for CXCR2, had no effect on cell motility and directedness in the presence of a dcEF (Fig. 4D). Similar to these results, the galvanotaxis of neural stem cells was also not mediated by SDF-1/CXCR4^47^, and hence we conclude that CXCR4 and CXCR2 are not involved in the galvanotaxis of BTICs.

### BTICs migrated toward the cathode in a three-dimensional extracellular matrix

Cell migration in a three-dimensional extracellular matrix represents a more physiological environment to study galvanotaxis^48^. However, very few galvanotaxis studies have been performed in 3D due to the technical difficulties^28^,^49^,^50^. By using a microfluidic device with a chamber geometry optimized for observing migration in 3D, we were able to conduct time-lapse experiments for 3D galvanotaxis. BTICs embedded in a 3D ECM consisting of hyaluronic acid (HA) and collagen were stimulated with a 1V cm^−1^ dcEF and compared to the observations in 2D. Hyaluronic acid is the most abundant ECM in the brain and has been shown to be critical in maintaining the homeostasis and recapitulate glioma invasion *in vitro*^51^,^52^. Glioma tumor spheroids embedded in a HA matrix functionalized with RGD peptides were previously utilized to elucidate the importance of mechanobiological regulation on cell motility, where glioma cells were found to invade the HA matrix with morphological patterns highly reminiscent of those seen in brain slices^52^. Although the brain is largely devoid of fibrillar proteins, collagen provides the necessary structural support and adhesion sites while keeping the HA/collagen composite within the range of stiffness and porosity found in the brain^4^. Various groups have highlighted the striking differences between 2- and 3D migration^53^,^54^. For example, to effectively migrate in a 3D matrix, glioma cells are required to degrade the ECM via matrix metalloproteinases and migrate through tortuous confinements with constant deformation and strong retraction forces, but neither of these are required in 2D^54^.

**Figure 5:**
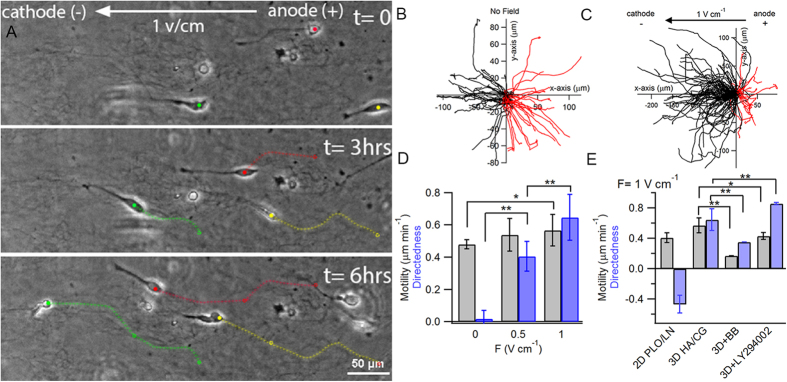
Galvanotaxis is a potent migration cue for BTICs in 3D. (**A**) Cells embeddded in ECM consisting of a mixture of 1 mg mL^−1^ collagen and hyaluronic acid exhibited a unique phenotype and migrated toward the cathode in the presence of a dcEF. Overlay of cell trajectories in the absence (**B**) and presence (**C**) of a dcEF indicated a strong cathodic response in the presence of an electric field. (**D**) Cell motility only significantly increased at 1V cm^−1^, whereas directedness progressively increased with increaseing field strnegth. (**E**) Cells exihibited opposite directional response in 2D and 3D. Inhibiton of myosin II with blebbistatin significantly down-regulated cell motility and directedness. However, inhibition of PI3K only slightly decreased cell motility but significantly increased cell directedness. *P < 0.05; **P < 0.01; Student’s t-test. Statistics were obtained from at least three independent experiments with at least 50 cells in each experiment. Error bars indicate standard deviation.

Here we observe significant differences in the galvanotaxis of BTICs between 2D and 3D. In 2D in the presence of an EF, migration of GBM612 cells is characterized by the formation of a small lamellipodium at the leading extended protrusion and the retraction of cellular processes at the trailing end (Supplementary Video S1). In contrast, in 3D lamellipodia were not observed and cells were devoid of trailing processes. Instead, cells migrated with a single long pseudopodia at the leading edge and underwent very dynamic cycles of extending and retracting protrusions similar to the migration of neural cells or glioma cells in brain slices^54^,^55^ (Supplementary Videos S6 and S7). Perhaps the most striking difference between 2D and 3D galvanotaxis of BTICs is the opposite directional response. In 3D in the presence of a dcEF, cells extended characteristic protrusions toward the cathode followed by contraction of the cell bodies to propel themselves (Fig. 5A,C), whereas on a PLO/LN coated surface, cells migrated toward the anode (Fig. 2E). Analysis of cell trajectories indicated that both cell motility and directedness significantly increased in 3D in the presence of a 1V  cm^−1^ field (Fig. 5D). On the other hand, fNPCs migrated toward the cathode in 2D and 3D, where both cell motility and directedness significantly decreased in 3D compared to 2D (Supplementary Fig. S2).

Pharmacological inhibition of myosin II and PI3K was selected to further investigate the mechanistic differences between 2D and 3D galvanotaxis. Myosin II has been previously shown to be critical for effective migration in restricted environments and to provide contraction to enhance adhesion stability^54^,^56^,^57^,^58^. On the other hand, the activity of myosin II must also needs to be delicately regulated for 3D migration as constitutive activation of myosin actually inhibited the invasion of glioma cells and increased the survival time in mouse models^59^. Treating cells in 3D with 50 μM of blebbistatin indeed showed a marked difference from the same treatment in 2D. While blocking myosin II in 2D had no effect on either cell motility or directedness, inhibition of myosin II in 3D significantly decreased both cell motility and directedness (Fig. 5E). PI3K, on the other hand, is involved in the galvanotaxis of many cell types in 2D including BTICs; however, its role in 3D galvanotaxis has never been reported. Treating cells in 3D with 50 μM LY294002 slightly decreased cell motility to 0.43 μm min^−1^ (p = 0.01) but surprisingly enhanced cell directedness to 0.86 (p = 0.004) (Supplementary Video S8). Taken together, these results show that galvanotaxis of BTICs in 3D is not mediated by PI3K but facilitated by a migratory mechanism that relies on myosin II contractility.

## Conclusion

We established that a dcEF is a potent directional migration cue for BTICs and fNPCs in both 2D and 3D. However, a number of divergences both phenotypically and mechanistically exist between different cell types and microenvironments. While all tested GBM sub-types exhibited anodic galvanotaxis in 2D, non-malignant fNPCs migrated toward the cathode, suggesting the relevance of malignant transformation on galvanotaxis. We further highlighted the complexity of galvanotaxis by showing the opposite in directional response and mechanistic differences between 2D and 3D galvanotaxis of BTICs, where the roles of myosin II and PI3K showed the greatest differences; myosin II is not required for galvanotaxis in 2D but necessary for effective 3D migration, whereas PI3K regulates the anodic response in 2D but enhances the cathodic bias in 3D upon inhibition. What determines the collective outcome in 2D versus 3D remains to be explored but very likely involves competing mechanisms as indicated previously^60^. Our results provide valuable insights in exploring the roles of endogenous EFs in pathogenesis and point out the importance of understanding how cell-ECM interaction may modulate galvanotaxis.

## Additional Information

**How to cite this article**: Huang, Y.-J. *et al.* Cellular microenvironment modulates the galvanotaxis of brain tumor initiating cells. *Sci. Rep.* **6**, 21583; doi: 10.1038/srep21583 (2016).

## Supplementary Material

Supplementary Video S1

Supplementary Video S2

Supplementary Video S3

Supplementary Video S4

Supplementary Video S5

Supplementary Video S6

Supplementary Video S7

Supplementary Video S8

Supplementary Information
