# Supplementary material for: Cellular microenvironment modulates the galvanotaxis of brain tumor initiating cells
Source: Sci Rep. 2016 Feb 22;6:21583. doi: 10.1038/srep21583 (PMC4761929; doi:10.1038/srep21583)
Supplement: Supplementary Information [file srep21583-s9.docx]

**Title**: Cellular microenvironment modulates the galvanotaxis of brain tumor initiating cells

**Authors:** Yu-Ja Huang^1,2^, Gwendolyn Hoffmann^1,2^, Benjamin Wheeler^1,2^, Paula Schiapparelli^3^, Alfredo Quinones-Hinojosa^3^, Peter Searson^1,2*^

**Supplementary Video S1**: Galvanotaxis of GBM612 on a PLO/LN coated surface

**Supplementary Video S2**: Galvanotaxis of fetal neural progenitors cells on a PLO/LN coated surface

**Supplementary Video S3**: Galvanotaxis of GBM612 treated with ROCK inhibitor

**Supplementary Video S4**: Galvanotaxis of GBM612 treated with PI3K inhibitor

**Supplementary Video S5**: Galvanotaxis of GBM612 treated with Erk1/2 inhibitor

**Supplementary Video S6**: 3D galvanotaxis

**Supplementary Video S7**: 3D galvanotaxis (close up view)

**Supplementary Video S8**: 3D galvanotaxis with PI3K inhibitor

**Supplementary Figure 1**. Trajectories of different brain cancer cell lines and fetal neural progenitor cells in the presence of a 1 V cm ^-1^ field.

**Supplementary Figure 2**. Summary of the galvanotaxis of fNPCs in 2D and 3D.


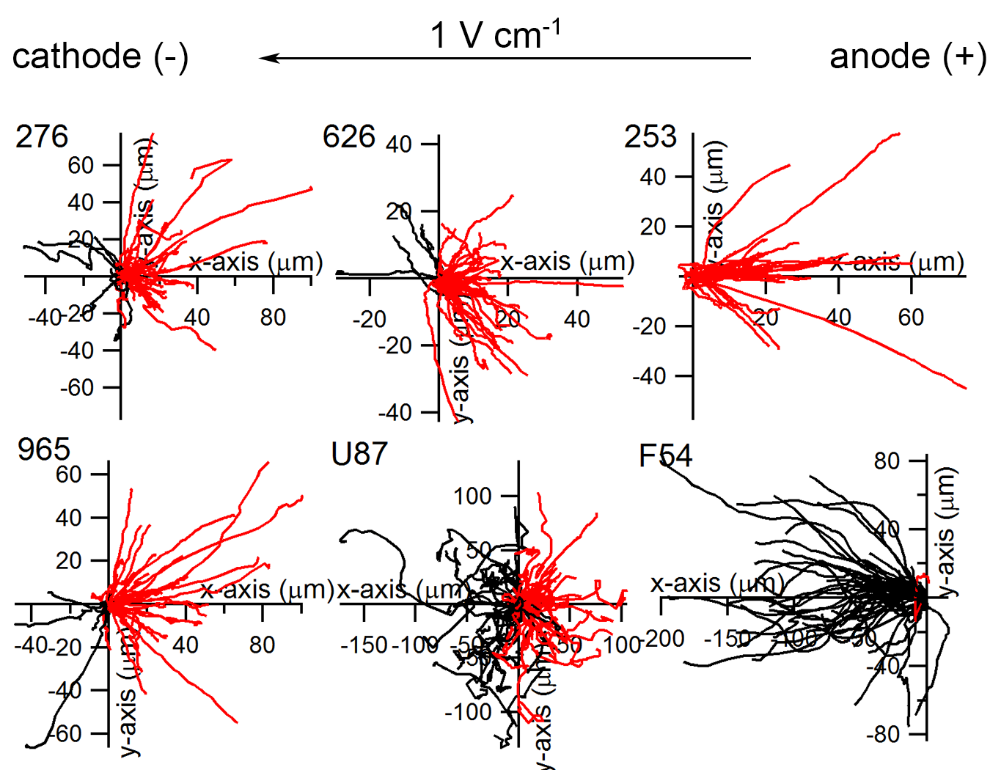


**Supplementary Figure 1. Trajectories of different brain cancer cell lines and fetal neural progenitor cells in the presence of a 1 V cm ^-1^ field.** BTICs of pronueral (276), mesenchymal (626 and 253), and classical (965) subtypes migrated toward the anode in the presence of a 1 V cm^-1^ electric field, whereas U87, an immortalized glioblastoma cells line, migrated randomly. Fetal neural progenitor cells (F54) exhibited opposite directional response than BTICs by migrating toward the cathode.


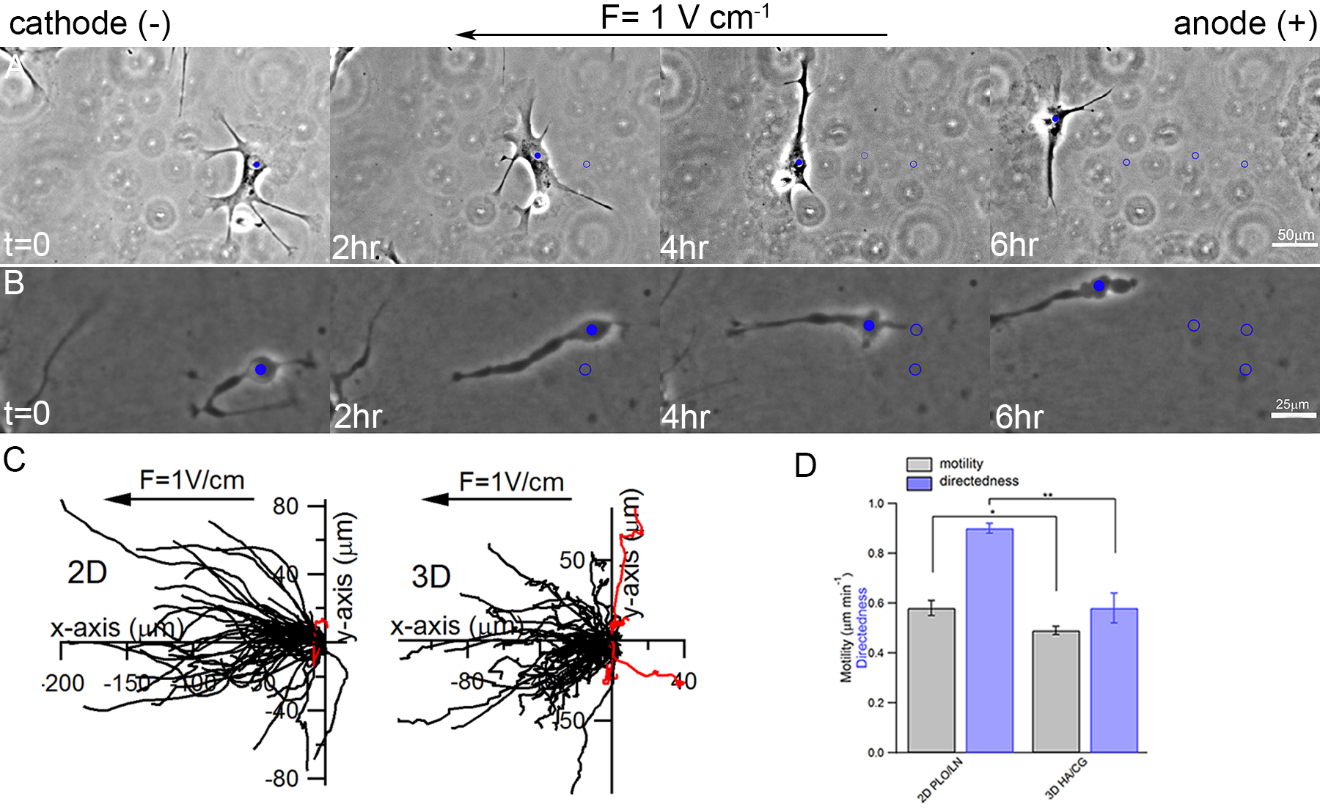


**Supplementary Figure 2. Summary of the galvanotaxis of fNPCs in 2D and 3D. (**A) Series of time lapse images of fNPC migrating on a PLO/LN coated surface in the presence of a 1 V cm^-1^ electric field; the cell extended a broad cathode-facing lamellipodium and migrated toward the cathode. The cell body also progressively aligned perpendicularly to the electric field as evidenced in the images correspond t=4 and 6 hours. The solid filled blue circles indicate the position of nucleus, whereas empty blue circles indicate the cell’s previous locations. (B) Time lapse images of a fNPC migrating in a 3D ECM composed of collagen and hyaluronic acid. In contrast to the broad lamellipodium observed in 2D, cells migrated mainly with a dominant protrusion and underwent frequent deformation. (C) Trajectories of fNPCs migrating in 2D (left) and 3D (right) indicated cell migrations are largely biased toward the cathode in both conditions. Cell trajectories, however, are more tortious in 3D comparing to 2D. (D) Summaries of cell motility and directedness in 2D and 3D. Both cell motility and directedness significantly decreased in 3D comparing to 2D.
